# Effect of Solute on Interfacial Properties and Micelle Structure of Dodecylbenzenesulfonate (DBS): Experimental and Molecular Dynamics Studies

**DOI:** 10.3390/ijms25010678

**Published:** 2024-01-04

**Authors:** Huang-Chin Hung, Gina S. Shreve

**Affiliations:** Department of Chemical Engineering and Material Science, Wayne State University, 5050 Anthony Wayne Dr., Detroit, MI 48202, USA

**Keywords:** molecular dynamic simulation, dodecylbenzenesulfonate, micelle structure, micellar solute localization

## Abstract

A combined experimental and molecular dynamic simulation approach was used to examine the structure and interfacial properties of solute-saturated micelles. The properties of dodecylbenzenesulfonate (DBS) micelles were examined in dodecane and benzene hydrocarbon systems. Pyrene fluorescence was used to determine the aggregation number of surfactant monomers in the micelle systems. Molecular dynamic (MD) simulations using energy minimization applying the CHARMm force field with the TIP3P model for water. Comparison of the DBS/benzene and DBS/Dodecane micelles equilibrium structures via radial distribution function (RDF) and probability distribution function (PDF) analysis indicates that the area per head group for the DBS/Benzene micelle interface is significantly larger than that of the DBS/Dodecane at the interface. It was also determined that benzene molecules can move freely within the micelle while dodecane is strictly confined in the core of the micelle. The increased interfacial area per monomer caused by the insertion of benzene also reduces the effectiveness of the surfactant, which has implications for use in various environmental applications. However, the DBS/benzene micelle can solubilize many more hydrocarbon molecules in one micelle with less surfactant monomer (i.e., lower aggregation number) per micelle due to the increased available packing positions within the micelle. This, in turn, increases the efficiency of the surfactant in real-world applications which is consistent with previous laboratory results. Understanding the differing solubilization characteristics of surfactants against various classes of hydrocarbons in single solute systems is a necessary step to beginning to understand their solubilization properties in the mixed waste systems prevalent in most surfactant enhanced remediation (SEAR) strategies.

## 1. Introduction

Understanding the molecular interactions between surfactants and hydrocarbons in micelles is the key factor for determining the effectiveness and efficiency of the surfactant in a system [1]. Previous studies comparing the effectiveness of surfactants in mixed waste systems yielded differing solubilization properties between these surfactants for varying classes of hydrocarbons [2,3,4]. One finding of those studies was that the solubility of aromatic compounds in the micelle is much higher than other types of hydrocarbons, yet the effectiveness against aromatic compounds is significantly lower than against straight-chain alkanes [2]. These results were consistent with early surfactant research indicating that surfactant/aromatic micelles might be bigger than surfactant/straight chain alkane micelles due to a larger area per head group in the hydrocarbon/water interface [1]. Such experimental results indicate that aromatic compounds localize differently in the micelle structure compared to straight-chain alkanes. Such differences may also be important for predicting the solubilization of various hydrocarbon species in mixed waste systems. 

While analytical techniques yield many important properties of micelle structure, they give little information on the underlying physical basis for the observed hydrocarbon solubilization properties and specificity. Molecular dynamic simulation of the structure of surfactant micelles yields important information on micelle size, structure, and other chemical properties. The correlation of micelle equilibrium structural parameters between simulation and laboratory-measured parameters, such as area per head group and aggregation number, provides validation of simulation results. 

Dodecane and benzene were chosen as solutes for the simulation studies since previous interfacial tension tests indicate that solubilization of dodecane and benzene in DBS micelles results in dramatically different micellar structures [2,3,4]. The large differences in molar volume, solubility and hydrophobicity between these solutes make them ideal candidates to examine the differences in micellar structures and solute specificity. DBS, which possesses a single aromatic ring in the head group that is similar to benzene and an eight-carbon hydrophobic tail, was chosen as the target surfactant due to its structural similarity to numerous petroleum-based surfactants, such as sodium dodecylsulfonate (SDS), a well-studied surfactant possessing a low aggregation number and stable, spherical micelles under a wide range of conditions [5]. Previous studies indicate that these results obtained here may be apprised with other studies of sodium dodecyl sulfonate systems which have been demonstrated to present slightly fewer constraints on molecular mobility within micelle structures than the DBS [6]. 

## 2. Molecular Dynamic Simulation Results

### Micelle Size and Area per Head Group

Micelle structures resulting from the CHARMm v31 simulations were approximated as ellipsoids in order to more accurately characterize their shape. Thus, instead of using only three parameters to fit the result as reported [7,8], six parameters are used to better describe their shapes. The following equation is used to fit the shape of the micelle and the coordinate of CD2 is used as the surface of the micelle since it is the separate point of the hydrophobic and hydrophilic portion of DBS.

(1) $$\frac{{\left(x-x_{0}\right)}^{2}}{a^{2}}+\frac{{\left(y-y_{0}\right)}^{2}}{b^{2}}+\frac{{\left(z-z_{0}\right)}^{2}}{c^{2}}=1$$

Equation (1) is for an ellipsoid with its longest axis aligned along the *x*-axis and the shortest axis along with *z*-axis. *x*_0_, *y*_0_ and *z*_0_ represent the centre of micelle. The length of the semi-axis along the *x*, *y*, and z directions are *a*, *b*, and *c*, respectively. For a prolate ellipsoid *b* = *c*, and an oblate, *b* = *a*. The eccentricity, e, is defined as Equation (2):

(2) $$e=\sqrt{1-\frac{c^{2}}{a^{2}}}$$

For a sphere, *a* = *b* = *c* and *e* = 0 while for a long needle like micelle *a* >> *c* and *e* = 1. The six parameters used for DBS/Benzene micelle were, in Å (19.6, 17, 15.8, −0.1, 0.2, 0.0, 0.548 for *a*, *b*, *c*, *x*_0_, *y*_0_, *z*_0_, e respectively) and (16.1, 13.7, 12.2, 0.0, −0.2, 0.2, 0.594 for *a*, *b*, *c*, *x*_0_, *y*_0_, *z*_0_, *e* respectively) for the DBS/dodecane micelles (Table 1).

To calculate the volume of a micelle with an ellipsoid shape *V_mic_* is straightforward with Equation (3):

(3) $$V_{mic}=\frac{4}{3}\pi abc$$

Assuming the micelle is a prolate ellipsoid and *b* = *c*, then equation 4 can be used to calculate the surface area of the micelle:

(4) $$A_{surface}=2\pi \left(c^{2}+\frac{ac}{e}\sin^{-1}e\right)$$

It is apparent from the simulation results that the Benzene/DBS micelle (*e* = 0.548) is more spherical compared to the DBS/Dodecane micelle (*e* = 594). Also, the DBS/Benzene micelle (*V_mic_* = 221 nm^3^) is substantially larger than the DBS/Dodecane micelle (*V_mic_* = 113 nm^3^). The area per head group can be calculated by dividing the total area of the micelle by the number of surfactant molecules. It shows the insertion of benzene molecules between DBS molecules greatly increases the area per DBS head group on the surface of micelles (A = 208.5 Å^2^) while the dodecane molecules pack within the core of micelle and result in a significantly smaller surface area per DBS molecule (A = 111.7 Å^2^). 

## 3. Discussion

**Figure 1:**
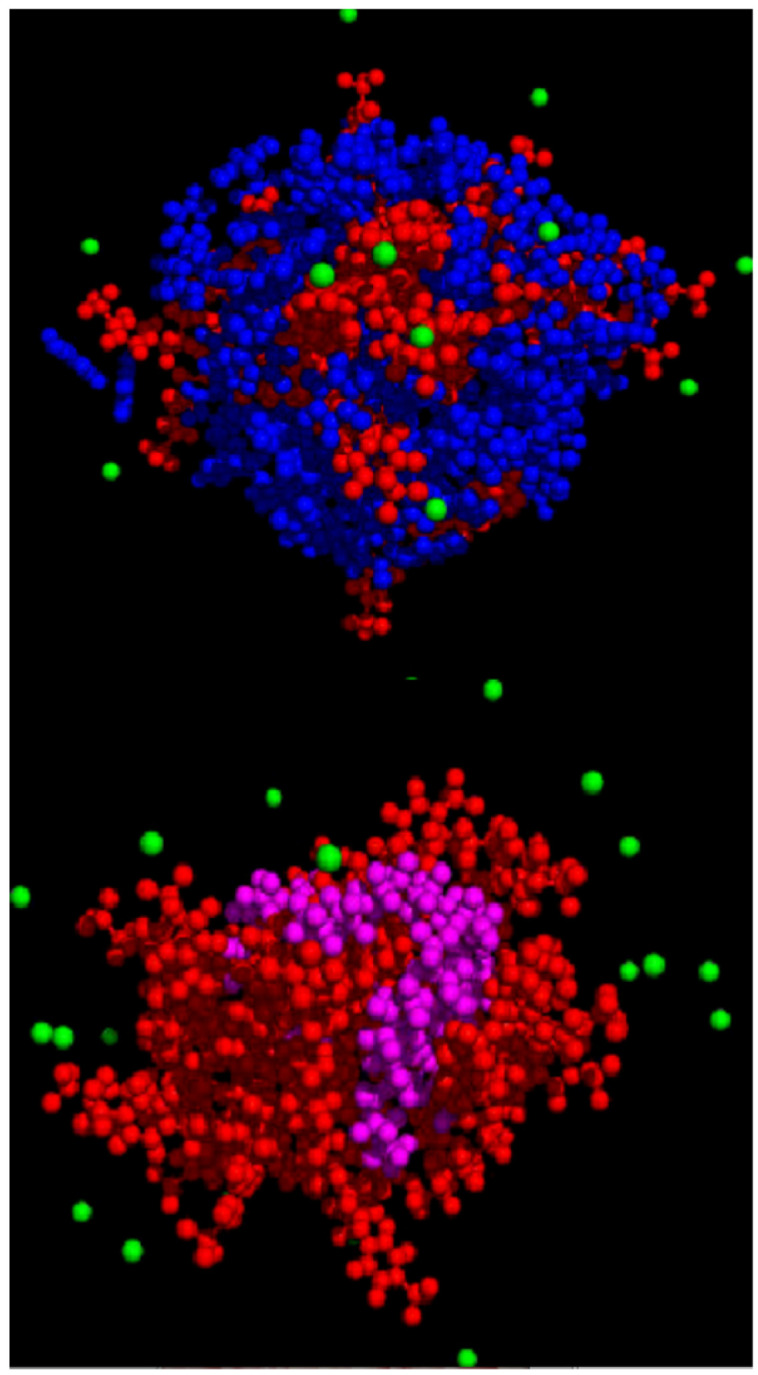
Simulation-based equilibrated Micelle Structure, top DBS/Benzene micelle, bottom, DBS/Dodecane micelle (•benzene, • DBS, • Dodecane, • Na+ ion).

Simulation output file coordinates of equilibrated structures are depicted as a space-filling model in Figure 1. Equilibrated structure results are consistent with previous reports that aromatic hydrocarbons tend to influence micelle structure by decreasing interfacial tension yielding a larger area per head group [1]. It is hypothesized that the aromatic compounds insert between the surfactant molecules on the interface and alter the interfacial properties between the two phases [1].

### 3.1. Localization of Water and Solutes within Micelle

**Figure 2:**
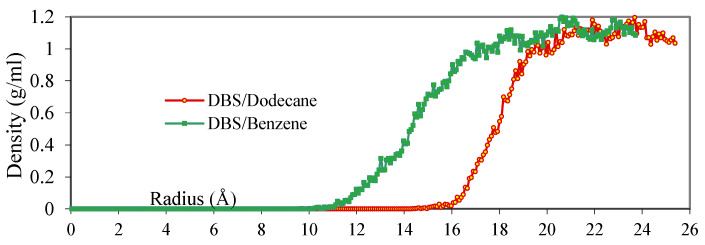
Water Density of the Micelle Systems.

Simulation results also indicate that water molecules penetrate approximately 4 Å deeper into the DBS/Benzene micelles than into the DBS/Dodecane micelles (Figure 2). The more hydrophilic benzene molecules at the outer layer of the micelle can “open” the micelle up for water molecules to penetrate the inner region. It is also noticed that the water/micelle interface is sharper for the DBS/Dodecane micelle (Figure 2). Since the interior of the DBS/Dodecane micelle is packed tightly with surfactant tail groups and dodecane molecules, which are highly hydrophobic, once the water molecules penetrate past the hydrophilic group of DBS they will meet strong resistance and are stopped quickly. In contrast, in the DBS/Benzene micelle structure, the benzene molecules are evenly distributed in the micelle, and the hydrophilic and hydrophobic regions of the micelle are not clearly distinguished.

**Figure 3:**
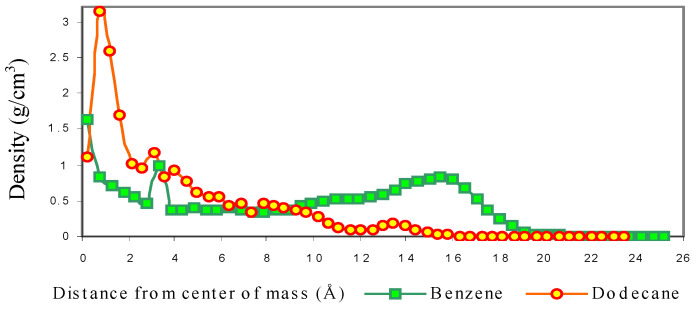
Localization of Solute based on Density within Micelle.

For the DBS/Dodecane micelle, the hydrophobic dodecane molecules remain near the centre of mass (Figure 3). It is also observed that the movement of dodecane molecules is strongly limited at the end of the simulation. The core of DBS/Benzene micelle is mainly occupied by benzene molecules due to its smaller molar volume. It can be observed that the density of benzene remains almost constant from 2–12 Å which means the benzene can move around within this hydrophobic core area of micelle (Figure 3). The density of benzene increases slowly from 12 Å to 15 Å, and this peak area represents where the majority of benzene molecules reside in the system in the outer layers of the micelles. From 17 Å the density of benzene drops lower than the average density within the density in the micelle, slowly decreasing to zero at around 20 Å. It is very interesting that the boundary of DBS/Benzene micelle is actually defined by a benzene/water interface, as there is no sharp boundary between micelle and solvent, while the DBS/Dodecane micelle is defined by the hydrophobic core area occupied by dodecane and DBS monomer tail groups, possesses a boundary that is very sharp, similar to that reported for micelles that are formed by pure surfactant molecules in the absence of solute [9].

### 3.2. Radial Distribution Function

**Figure 4:**
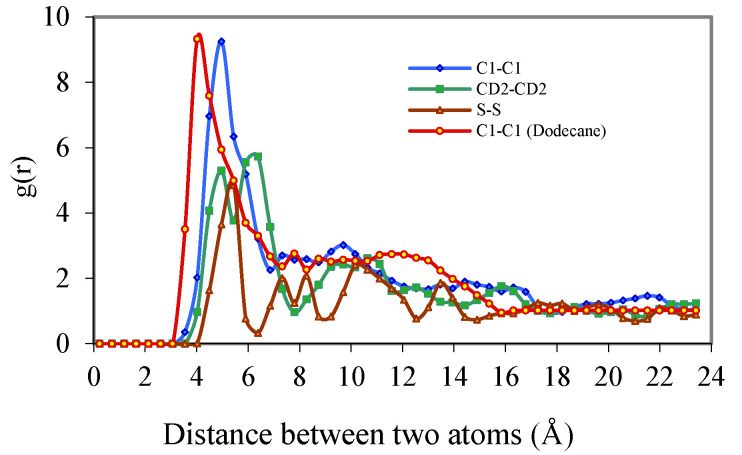
Radial Distribution Functions of DBS/Dodecane Micelle.

The radial distribution function (RDF) is calculated from the pair distribution function *g*_2_ (*r*_i_, r_i_), or simply *g*(*r*), and represents the probability of finding a pair of atoms a distance r apart, relative to the probability expected for a completely random distribution of atoms at the same density [10]. This was calculated using standard approaches [11]. Figure 4 shows the radial distribution functions (RDF) of DBS/Dodecane micelle. The RDF value for C1, indicates a significant peak at 5 Å and a minor peak at 8 Å, which means 5 Å is the preferred distance between C1 atoms among the surfactants indicating a highly ordered packing pattern. However, the RDF of CD2 shows two preferred packing patterns with a distance of 5 Å, which is the same position as the C1 peak, and a larger distance of 6.4 Å. These two peaks show about the same intensity indicating that two types of packing patterns of aromatic rings are favored when the two aromatic rings are close to each other. The first type represents when the aromatic rings line up to each other parallel, like two plates on the table. This packing pattern provides the minimum distance for the two aromatic rings.

**Figure 5:**
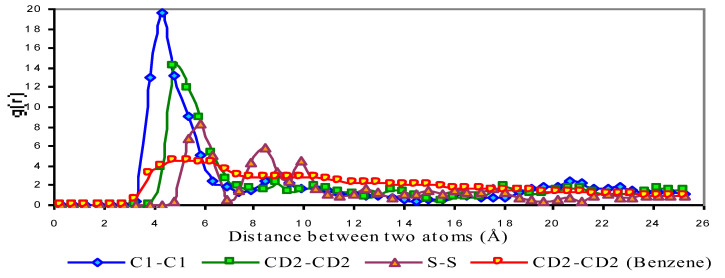
Radial Distribution Functions of DBS/Benzene Micelle.

This distance is 5 Å, when two surfactants are perfectly aligned, therefore, 5 Å is the ideal distance between any atoms of the same type for two different surfactant monomers. The second packing type is two aromatic rings facing each other with an ~70˚ angle, like an open chest and its’ lid. This type of packing pattern provides a slightly longer distance (6.4 Å) between two surfactants. A reasonable explanation for this is that the tails of the surfactants retain a high strain within the center of the micelle which prevents the aromatic rings from packing themselves at the closest distance resulting in the occurrence of a secondarily favoured packing pattern. The ratio of these favoured distance packing patterns may be an index of how much strain the tail of the surfactant experiences when it is positioned in the centre of the micelle with other tails. Sulfur atoms show a packing pattern of 5 Å as predicted, however, another slightly higher packing distance (5.6 Å) is present as well (Figure 4). The two peaks are not well separated, possibly because the negative charge that sulfur atoms carry drives the two sulfur atoms a bit farther apart forming a wider spectrum. The DBS/dodecane micelle RDF (Figure 5) also shows a very broad peak from 4 to 14 Å suggesting no specific favored packing pattern. 

Figure 5 shows the RDF of the DBS/Benzene micelle. For C1 atoms, similar to the DBS/Dodecane micelle, the RDF yields one significant peak at 4.2 Å indicating a single favoured packing pattern. As in the earlier discussion water penetrates deeper into the DBS/Benzene micelle and is likely the cause of the tighter packing pattern for C1 atoms as the excess water molecules neutralize the negative charge of DBS and result in a shorter distance between each C1 atom. 

For CD2 atoms the RDF (Figure 5) indicates two possible packing patterns similar to the DBS/Dodecane micelle, and these two peaks appear at 4.8 Å and 5.8 Å. Compared to DBS/Dodecane micelle these packing distances are a bit shorter for the first peak but much shorter for the second peak. The reason for the reduced distance of the first possible packing pattern might be the same as C1 atoms. However, the greatly reduced distance for the second pattern implies less strain in the centre of the micelle and surfactants are allowed to line up much closer with a smaller angle. For sulfur atoms, the largest RDF peak is at 5.6 Å followed by a much smaller peak at 8.4 Å. The first distance between sulfur atoms is a bit shorter compared to DBS/Dodecane micelle; it will be shown later that unlike dodecane molecules, which tend to stay in the core of the micelle, many benzene molecules can be packed at the surface of the micelle, and these benzene molecules prevent the surfactant’s hydrophilic head group from lining up tightly on the surface of the micelle. The second distance is much farther than that for the DBS/Dodecane micelle since the sulfur atom is very hydrophilic and water molecules penetrate deeper compared to DBS/Dodecane micelle. The effect of dissociation from water molecules, the same force that drives sodium ions away from sulfonic groups, is stronger and drives the two sulfur atoms apart. Unlike dodecane molecules in the DBS/Dodecane micelle, the benzene molecules show no significant packing patterns or favoured packing distance within the DBS/Benzene micelle suggesting that benzene is free to move around within the micelle with little constraint.

### 3.3. Probability Distribution Function 

**Figure 6:**
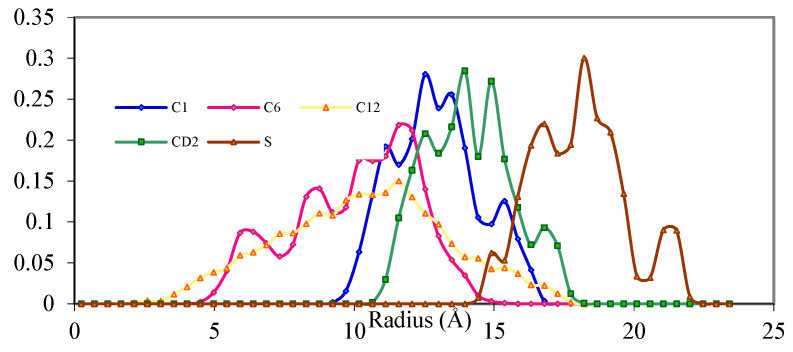
Probability Distribution Function of DBS/Dodecane micelle.

The probability distribution function (PDF) is calculated from the density function of the resulting equilibrated micelle structure and represents the probability of finding a certain atom at a specified distance (radius) away from the centre of mass (COM) [12]. The PDFs were normalized by the total number of atoms so that the area under each peak represents the probability of finding the atom at that distance. From the PDF of the DBS/Dodecane micelle, there are three favoured positions for C1 atoms, 11 Å, 13 Å and 15 Å radial distance away from the centre of mass (COM) (Figure 6). The PDF of CD2 is almost the same shape compared to C1 because these two atoms are linked. The major difference is that the PDF of CD2 is shifted by around 1.7 Å farther away from the COM (Figure 6). Compared to the standard bond length of C1-CD2 (1.49 Å) between the hydrophilic and the hydrophobic parts of the DBS, an extension of the bond length is observed. The sharp change of hydrophobicity in this region may exert stress along the bond of C1-CD2. Also, a much wider range of positions from 5 to 15 Å are available compared to C1. In the centre of the DBS/Dodecane micelle the available space is fully filled with folded tail groups of DBS and dodecane molecules and the C6 atom (the middle atom of the tail group) cannot remain at a fixed position as the folding of tails renders all carbon atoms randomly.

In comparison to C6, the C12 atom has a wider range of positions available in the micelle since it is the end group of the surfactant tail, hence, it appears at the core (2 Å) as well as the surface of the micelle (17.8 Å) (Figure 6). Unlike other carbon atoms of the tail group, C12 possesses a weak dipole and is more hydrophilic than other carbon atoms thus explaining why C12 can stay closer to the surface of micelle compared to the other carbon atoms.

The PDF locating the sulfur atom defines the outer layer of the micelle that forms by highly hydrophilic atoms. A range of 14.5 Å to 22 Å from COM is observed for the sulfur atom and that is around 4 Å distance from the CD2 atom. Since the aromatic part of DBS is not flexible, the distance between CD2 to sulfur should be 4.49 Å, theoretically. The difference observed might be due to deformation of the aromatic ring or extension of the S-C bond, further evidence that stress is present in the hydrophilic head group of the DBS/dodecane micelle. 

The behaviour of dodecane within the micelle is examined through the PDF of C6 and C1/C12 atoms. Compared to the C12 of DBS, the C6 and C1/C12 atoms both penetrate much deeper into the micelle. A much wider range of positions is also observed for both C6 and C1/C12 atoms compared to C12 of DBS due to the absence of hydrophilic parts in dodecane thereby allowing dodecane to fold itself in any orientation in the centre of the micelle. Another interesting observation is that C1/C12 (the end methyl group of dodecane) prefers positions closer to the surface of the micelle compared to C6 or other carbons in the middle of the chain. This behaviour was observed in terms of hydration number reported previously with SDS and DBS [13,14] and can be explained by the existence of a weak dipole at the end of the methyl group.

Hence, the behaviour of the DBS/Dodecane micelle is more similar to the micelle formed by pure surfactants in the absence of solute [9,10,11,12,13]. Since the dodecane molecules are highly constrained within the core of the micelle, the local dielectric constant likely varies rapidly from the outer layer of the micelle to the core, with an abrupt change at the position of the juncture between hydrophilic and hydrophobic functional groups of the surfactant. Evidence of this rapid decrease in hydrophilic property is the deformation of the surfactant structure due to the stress between hydrophilic and hydrophobic regions as the surface of the micelle remains very sharp similar to micelles formed by pure surfactants [13,14]. 

**Figure 7:**
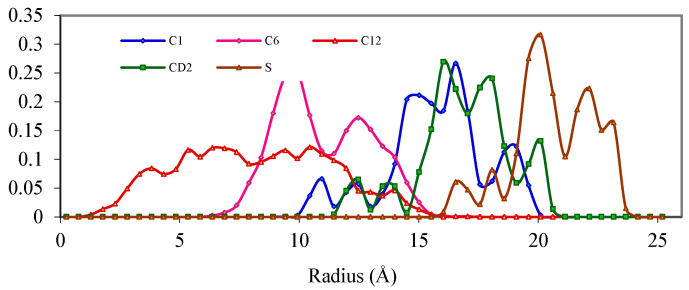
Probability Distribution Function of DBS/Benzene micelle.

In contrast, the interface of DBS/Benzene micelle is not clearly defined because the hydrophilic property reduces slowly and continuously from the outer layer into the core due to the “buffer” effect. The evidence is that the surfactant in DBS/Benzene shows little constraint and tends to retain its conformation with minimum potential energy. Examination of the PDF resulting from the equilibrium structure indicates that the C1 atom of DBS in the DBS/Benzene micelle system shows a wide range of positions from 10–20 Å, although most C1 atoms appear at a range of 13–20 Å (Figure 7). These values show a less ordered structure of C1 in DBS/Benzene micelle compared to DBS/Dodecane micelle indicating the C1 atom has access to more positions. It is also apparent that these C1 atoms stay farther from the COM than their counterparts in the DBS/Dodecane micelle. Benzene molecules expand the micelle more than dodecane molecules by allowing water molecules to penetrate deeper into micelles. Since water and benzene are both small molecules with little stereo effect, they can move freely resulting in a more flexible and less structured micelle interior.

Comparing the PDF of CD2 and C1 (Figure 7) it is apparent that they are almost in the same range; the CD2 atoms are only ~0.5 Å away from C1 compared to the bond length of 1.49 Å. Since it is impossible for the bond to be compressed to 0.5 Å, C1 and CD2 must be lying at almost the same “shell” corresponding to the centre of the micelle, which implies that the entire aromatic parts are lying on the surface perpendicular to the micellar surface. Assuming the bond length of C1-CD2 remains at roughly 1.49 Å, the angle between the radial axis to the aromatic plane would be around 70.5 º while the aromatic ring of DBS lines totally along with the radial axis (0°) in DBS/Dodecane micelle. This behaviour again proves that there is less strain between the hydrophilic parts and hydrophobic parts of DBS in the presence of benzene molecules because the aromatic rings are almost equal in terms of hydrophilic properties and all aromatic carbon should have lines in a similar radial position concerning COM if no excess force is exerted. 

The position of C6 atoms in DBS/Benzene is highly ordered unlike the C6 atoms in DBS/Dodecane micelle and it ranges from 7–15 Å (Figure 7); this fact suggests that the orientation of the alkane chain among C1–C6 atoms is less random. By examining their configuration, it can be determined that these alkane chains stay in standard sp^3^ configuration of minimum energy. The C6 atom is even more ordered than the C1 atom of the same molecule as the peak of C6 is narrower because of the absence of water molecules. 

**Figure 8:**
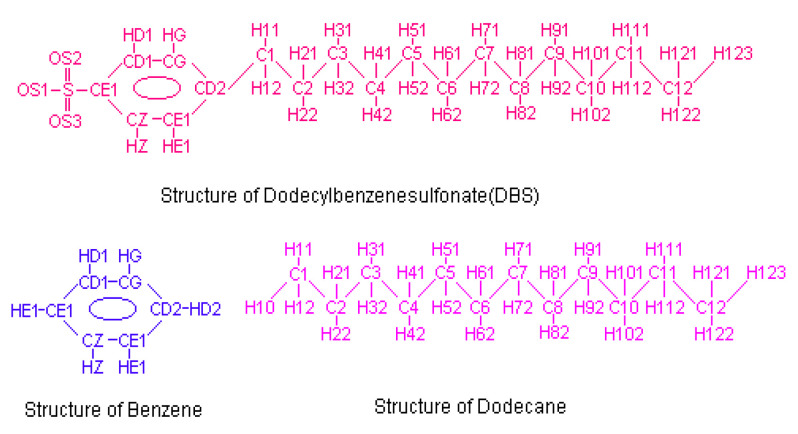
Structures and numbering of molecules used for MD simulation.

At C12, the end carbon atom of the tail, it appears the folding process has equilibrated rapidly rendering the position of C12 to be highly random with a range of 2–14 Å (Figure 8). Compared to the DBS/Dodecane system, it can be observed that C12 reaches farther to the surface in the DBS/Dodecane micelle (18 Å). The position that C12 can occupy is restricted to the hydrophobic area and water molecules penetrate deeper into the DBS/Benzene micelle, as a result, the hydrophobic zone is shrunk and the distance that C12 can travel is shorter relative to DBS/Benzene micelle.

The position of benzene molecules is indicated by the PDF of the CD2 atom of benzene. Benzene molecules can travel everywhere inside the micelle and even outside the micelle for a limited distance (0–21 Å from COM). Although the benzene molecules can access the core of the micelle, most of the benzene molecules stay at 14–16 Å, which is the boundary of the micelle. Further examination of the result of these molecular dynamics simulations shows that benzene molecules play the role of a “buffer” molecule due to its higher dielectric constant that allows water to penetrate deeper into the micelle, resulting in an increase in surface area per head group that is a combined result of the presence of both water molecules and benzene molecules [14,15,16].

## 4. Materials and Methods

Experiments show that the formation of micelles can take up to milliseconds (ms) in nature [17,18]. Since the computational resources available for this study yielded only up to several nanoseconds [7,8], rendering the surfactant monomer, hydrocarbon and water molecules in a totally random fashion was impractical, therefore molecular dynamic (MD) simulations were performed with initial coordinates rendered based on geometrical considerations using previously determined interfacial area [2] and monomer aggregation number to extract a satisfactory result within these time constraints. The coordinate of each atom for a micelle system was carefully rendered in a spherical control volume with the surfactant hydrophilic group facing outward and the hydrophobic tail within the interior. The amount of molecules that form one micelle is based on the experimental results [2,3], energy minimization was performed to remove bad contacts between atoms and the molecular dynamics simulation using CHARMm v31 [19] force field was conducted (NPT, 2ns). No additional constraints were applied throughout the processes. 

### 4.1. Pyrene Fluorescence Determination of Aggregation Number

Prior to simulation, the aggregation number of each micelle was experimentally determined by pyrene fluorescence quenching experiments. The micellar solubilization ratio (MSR) defined as moles of hydrocarbon solute per mole of surfactant for both benzene and dodecane solute was obtained from gas chromatograph (GC) measurements [3]. Thus, the composition of each micelle (DBS/benzene and DBS/Dodecane) was clearly defined. The pyrene fluorescence probing method involves the excitation of micelle-solubilized pyrene and the study of the fluorescence decay curves [20]. Pyrene is a hydrophobic molecule that emits fluorescence at a wavelength of 385 nm when excited by a hydrogen laser (330 nm). Because of the different decay rates of pyrene molecules, the relative emission of pyrene fluorescence (I/I_max_) differs as a function of pyrene concentration and the ratio of micelle concentration to pyrene concentration can be obtained from the curves of relative emission. 

Pyrene (crystals, SIGMA, Burlington, MA, USA), sodium dodecylbenzenesulfonic (crystals, SIGMA, Burlington, MA, USA), n-dodecane (99%, SIGMA, Burlington, MA, USA) and benzene (99%, Fisher Chemical, Waltham, MA, USA) were used as received. De-ionized water was prepared in the lab using a Millipore deionization system. Dissolution of the probe in the micelle solutions was accomplished by stirring the solution at room temperature (25 °C), in a tube with the interior covered by a thin film of the probe. Micelle solutions were prepared and equilibrated so that the micelle was fully loaded with hydrocarbon; this was accomplished by introducing extra hydrocarbon into the DBS solution, shaking it for 8 h followed by centrifugation (Hyland, Westlake, OH, USA) at 4000 rpm for 15 min. The solution was allowed to stand for 12 h for better separation of the two phases and the aqueous micellar phase was removed for further analysis. The DBS/dodecane micelle and DBS/benzene solutions were prepared using dodecyl benzenesulfonate at a concentration 1000 mg/L higher than their critical micelle concentration value of 8 mmol/L [21]. Pyrene was added to the solution at concentrations of 22.25, 44.5, 89 and 178 mol/L. The solution was sealed and frozen by liquid nitrogen, then the air was pumped out. The process was repeated at least three times to ensure all air was removed from the sample solution. Fluorescence signal data points were averaged over sets of 10 individual signal points utilizing a pulse hydrogen laser yielding 300 fluorescence values representing 3000 individual measurements. Since the environmental noise contributes a large fraction of the signal (10–20%), a blank sample with no pyrene was measured to eliminate the noise. The relative intensity curves can be represented as a straight line when the time is long enough and the following relationship holds:

(5) $$\ln(\frac{I(t)}{I(0)})=k_{0}t-R$$

where *R* is the ratio of micelle concentration [*M*] to pyrene concentration [*P*]. Plot ln[*I*(*t*)/*I*(*0*)] vs. *t*, the experimental data can be fitted with a straight line and *k*_0_ and *R* will be obtained. The aggregation number can be obtained by the total concentration of surfactant *C* and the *cmc* of the micellar system:

(6) $$n=\frac{C-cmc}{\left[M\right]}=\left(C-cmc\right)\frac{R}{\left[P\right]}$$

Experiments were conducted using 3, 9, 18 and 36 ppm of pyrene to determine an average of aggregation number *n* (Table 2). No systematic error relating to pyrene concentration was observed indicating that the introduction of pyrene doesn’t interfere with the micelle formation during the experiments. The average aggregation number of DBS/Benzene micelle at 1 atm and 300 K was determined to be 17 and the aggregation number of DBS/Dodecane micelles is 22 (Table 2).

### 4.2. Prediction of Micellar Structure with Molecular Dynamics Simulation Method

CHARMm Program version 27b was used for the minimization and simulation of the micelle and solute systems. Force field calculations used the par_all22_prot_lipmod.inp routine in CHARMm 27b [19]. Some of the parameters were missing thus an estimate of values was derived using Gaussian™ vGO3. To generate a set of coordinates of molecules that represent a spherical micellar structure, all the coordinates were rendered inside the spherical control volume with an initial orientation. Reference points were generated on the surface of the sphere then the rest of the atoms of the same molecule were built based on those points. The area each reference point occupies was estimated by simply dividing the total surface area by the number of references. To represent a molecular structure based on the reference points, the coordinates of each atom corresponding to the reference point were expressed as a set of 3 mathematical functions based on the bond length, bond angle and the distance between the atom to the centre of the spherical control volume. Finally, the coordinates of the entire molecule were assigned based on these functions and the reference points. Water molecules were generated in a cubic control volume with a radius of 23 Å with the spherical void reserved for the micelle structure. The dodecane structure was created by simply extending its length to 12 carbon atoms from octane. The structure of the DBS Monomer was created by inserting an aromatic ring into the sulfonic octane and extending the tail to 12 carbon atoms. Figure 8 illustrates the structure and atom names of DBS, Benzene and Dodecane molecules. 

The TIP3P intermolecular potential 3P was used to model force functions for water [22]. For both the micelle/solute systems, the initial spherical volume reserved for micelle was created with a radius of 21 Å as the cutoff radii for electrostatic and vanderWaaals interactions. The hydrophilic groups of DBS were immersed in the water layers. Once the coordinates of the whole system were generated, the system was minimized to eliminate bad contacts as well as some strain within bonds, angles, and dihedrals. Each minimization consists of 1000 steps of the steepest descents (SD) method followed by 1000 steps of Newton-Raphson (NR) method. Once the whole system was minimized at 298 K, a subsequent minimization was conducted at 300 K with time steps as 0.001 ps and 300 steps. To better approximate previously published experimental results [2] with the molecular dynamic simulation, NPT (constant number of molecules, pressure at 1 atm and temperature at 300 K) was chosen to simulate because all the experiments, used to guide knowledge-based construction of input file structures, were conducted under these conditions. The full CHARMm NPT simulation was then performed with settings of Pmass 400, Pgamma 20 and Tcoupling 5.0 with results sampled at every 5 ps (5000 steps). 

## 5. Conclusions

The results obtained agree with previous studies indicating that benzene molecules can partition at the interface or the surface of the micelle between hydrophilic and hydrophobic phases. Detailed comparison of the equilibrated micelles/solute structures for the DBS/Benzene and DBS/Dodecane micelles, shows that the area per head group on the DBS/Benzene interface is larger than those for the DBS/Dodecane interface. Further examination indicates that a highly compact micelle is formed for the DBS/Dodecane system while a large, loosely packed micelle is formed for the DBS/Benzene system. It was also determined that benzene molecules can move freely within the micelle while dodecane is strictly confined in the core of the micelle. This result agrees with the previous studies indicating that benzene molecules can partition at the interface or the surface of the micelle between hydrophilic and hydrophobic phases [23,24,25]. The increased surface, or interfacial, area per surfactant head group caused by the insertion of benzene also reduces the effectiveness of the surfactant. However, the DBS/Benzene micelle can solubilize many more hydrocarbon molecules in one micelle with less surfactant monomer (i.e., lower aggregation number) per micelle due to the increased available packing positions within the micelle, which in turn increases the efficiency of the surfactant. This is also consistent with laboratory results obtained using interfacial tensiometry [2].

In conclusion, by combining experimental techniques and molecular dynamics simulation methods, the structure, and interfacial properties of solute-saturated micelles were studied in great detail. The solute effect of the micelle was determined using both simulation and experimental methods. These methods allowed the determination of detailed structural information for these surfactant/solute systems and will be useful in future work designed to extract more detailed thermodynamic properties from the MD simulation results as well as to extend these studies to additional surfactant and mixed solute systems of interest. The equilibrated micellar structure of DBS/Benzene is shown in the Cover Art for DBS/Benzene (blue) and DBS/dodecane (purple) remaining atoms are colored as follows: sodium ions (green), DBS surfactant (red).

## Figures and Tables

**Table 1:** Ellipsoid Configuration Parameters fit to Simulation Results.

Micelle Type	*a* (Å)	*b* (Å)	*c* (Å)	*x*_0_ (Å)	*y*_0_ (Å)	*z*_0_ (Å)	*e*
DBS/Dodecane	16.1	13.7	12.2	0.0	−0.2	0.2	0.594
DBS/Benzene	19.6	17	15.8	−0.1	0.2	0.0	0.548

**Table 2:** Aggregation Number Calculated from Pyrene Fluorescence Quenching Tests in Dodecylbenzenesulfonate (DBS) micelle.

Pyrene Concentration (mg/L)	3	9	18	36	Average	Standard Deviation
Dodecane/DBS	19.3	20.5	26.3	21.3	21.85	1.78
Benzene/DBS	19.9	17.1	14.1	15.3	16.6	1.46

## Data Availability

Data are contained within the article.
